# Intriguing Interaction of Bacteriophage-Host Association: An Understanding in the Era of Omics

**DOI:** 10.3389/fmicb.2017.00559

**Published:** 2017-04-07

**Authors:** Krupa M. Parmar, Saurabh L. Gaikwad, Prashant K. Dhakephalkar, Ramesh Kothari, Ravindra Pal Singh

**Affiliations:** ^1^Bioenergy Group, Agharkar Research InstitutePune, India; ^2^Department of Biosciences, Saurashtra UniversityRajkot, India

**Keywords:** bacteriophage, genomics, next-generation sequencing, transcriptomics

## Abstract

Innovations in next-generation sequencing technology have introduced new avenues in microbial studies through “omics” approaches. This technology has considerably augmented the knowledge of the microbial world without isolation prior to their identification. With an enormous volume of bacterial “omics” data, considerable attempts have been recently invested to improve an insight into virosphere. The interplay between bacteriophages and their host has created a significant influence on the biogeochemical cycles, microbial diversity, and bacterial population regulation. This review highlights various concepts such as genomics, transcriptomics, proteomics, and metabolomics to infer the phylogenetic affiliation and function of bacteriophages and their impact on diverse microbial communities. Omics technologies illuminate the role of bacteriophage in an environment, the influences of phage proteins on the bacterial host and provide information about the genes important for interaction with bacteria. These investigations will reveal some of bio-molecules and biomarkers of the novel phage which demand to be unveiled.

## Introduction

Innovations in next-generation sequencing (NGS) technology and the decline in the sequencing cost have triggered a revolution to gain an understanding into the diversity, structure, and function of complex microbial processes (Vlahou and Fountoulakis, 2005). NGS has enhanced our concept of various influences of microbes in maintaining equilibrium in the environment and accentuating the function of diverse hosts including humans (Li et al., 2008). NGS has expedited the interpretation of microbiome using techniques, such as metagenomics, metatranscriptomics, metabolomics, proteomics, and single cell genomics. Apart from sequencing, bioinformatics and statistical tools also represent a significant role in sequence assembly, alignment, binning, and annotation. Software of bioinformatic assists in decoding the identity, abundance profile, genetic composition and functional channels of an organism or for a microbial community (Meyer et al., 2008; Glass et al., 2010). Genomics deals with sequencing of the whole genome of a distinct organism whilst metagenomics study a pool of genomes of a community of different populations (Handelsman, 2004). According to the central dogma, the flow of genetic information in a cell is from DNA (genome) to RNA (transcriptome) and then it is translated into proteins (proteome) (Crick, 1970). Genomics elucidates presence of the gene in an organism, while transcriptomics provides the information about the genes that are actively expressed in an organism and proteomics study the structure and function of every protein in an organism. A novel technique called single cell genomics takes in record information of the sequence from an individual cell which procures a better degree of accuracy in cellular differences and a finer understanding into the function of a particular cell in an ecosystem (Eberwine et al., 2014). However, metabolomics includes the analysis of metabolites of an organism and these results may vary from genomics and transcriptomics data as they are influenced by surrounding environments (Apel and Hirt, 2004). Apart from sequencing, bioinformatics and statistical tools assist in sequence assembly, alignment, binning and annotation.

**Figure 1:**
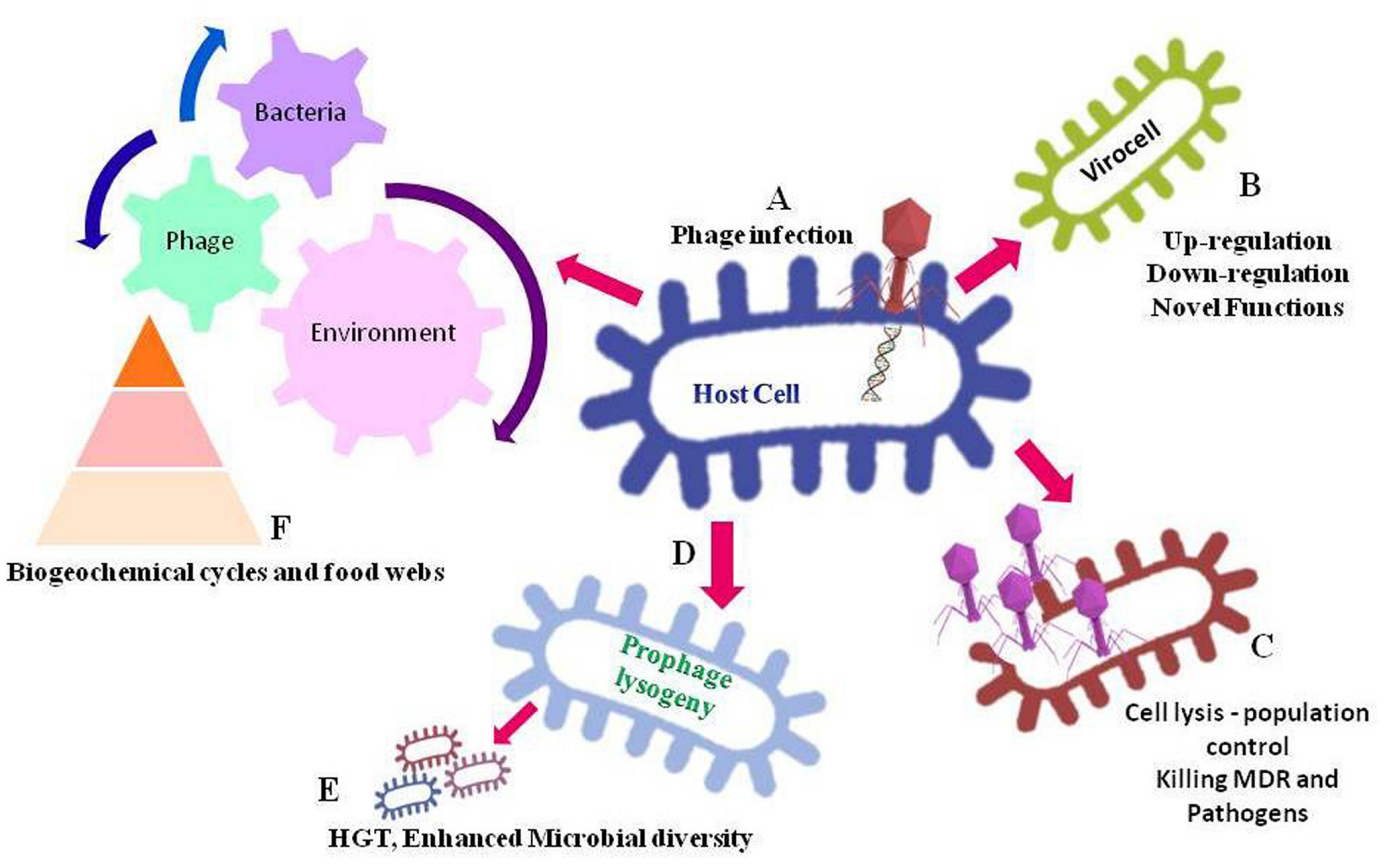
**Significance of bacteriophages in regulating environment**. In this figure, **(A)** depicts infection and insertion phage DNA into the host cell. After the phage infection, the host cell may get converted into a virocell **(B)**, containing vAMGs that leading to an altered regulation or novel functions in bacterial host cell. The phage infection leading to lytic cycle **(C)** results into lysing host cell hence, controlling cell population. Infected cell leading to a lysogeny cycle **(D)** may contain phage genome into the bacterial genome, which can lead to an increased microbial diversity because of horizontal gene transfers- HGT **(E)**. Also, the dead debris of bacteria as a result of phage lysis enters the food-web and biogeochemical cycles **(F)**, as a result the nutrients get re-circulated in the ecosystem.

Whilst, the information about bacteria present on earth is better understood, data regarding viruses particularly bacteriophages (henceforth called phages) is still in its infancy. Phages are the most abundant and diverse group of viruses found on Earth (Short and Suttle, 2005). Interactions between the bacterial host and phage have significanty played an role in biogeochemical cycles, regulation of the microbial community structures and governing the microbial populations (Figure 1). Recent surveys have documented the capacity of phages in maintaining the stability of microflora in the human gut (Minot et al., 2011) and regulation of pathogen and multidrug resistance (MDR) in the environment (Parmar et al., 2017). In bacteria, 16S rRNA genes and several house-keeping genes serve as a biomarker which facilitate their identification, whereas, there is an absence of biomarker gene among phages, which poses as a hindrance for identification of phages and hence the phage database is quite insufficient (Edwards and Rohwer, 2005). Addressing the challenge to design a biomarker for phages may uncover new avenues in better understanding the virosphere. Plaque assay being a culture-dependent technique isolates a specific phage against a bacterial host. Subsequently, their identification can be worked out using phenotypic characteristics and sequencing (Sanchez et al., 2015). In an approach to improve a comprehensive insight into uncultivable phage and their interaction with the bacterial host, this review summarizes different NGS techniques and the bioinformatics tools that are applied to evaluate such data.

## Bacteriophages and their interaction with bacterial host

Phages are in 10:1 ratio with bacteria on Earth, though viral DNA corresponds to only 0.1% of total DNA among microbial communities (Qin et al., 2010). Phages are ubiquitous in the environment and are found abundantly where bacterial hosts thrive. Mostly phages flourish in oceans, soil, wastewater treatment plants, hot-water springs and animal gut (Wommack and Colwell, 2000; Prigent et al., 2005; Prestel et al., 2008; Srinivasiah et al., 2008). Phages are classified on the basis of their size, structural composition, genome organization and on the host it infects (Ackermann, 2009). Electron microscopy assists in studying phenotypic characteristics of phage on the basis of the size and shape of head, tail and tail fibers. The most abundant phages in the environment are dsDNA belonging to order *Caudovirales* (Weinbauer and Rassoulzadegan, 2004). *Caudovirales* are furthermore classified into *Podoviridae* having a short tail, *Siphoviridae* with long non-contractile tail and *Myoviridae* possessing a long contractile tail. Every phage is specific toward a particular bacterial host and may have a narrow or broad host range depending on its infection capability. Hosts provide the enzymatic machinery for the phages to replicate and multiply by infecting the most active (exponentially growing) bacteria as implied by “kill the winner” hypothesis (Rodrigue et al., 2009). Phages undergo two types of life cycles, (1) in the lytic lifecycle, phage injects own DNA into a host cell and multiply by manipulating host replicating machinery. After replication, phage cleaves the host bacterial cells releasing progeny virus particles into the environment. Whereas, in (2) lysogeny lifecycle, phage DNA integrates into the bacterial genome, replicates their DNA along the bacterial genome and transfer on to the progeny of host cells (Bertani, 1951).

**Figure 2:**
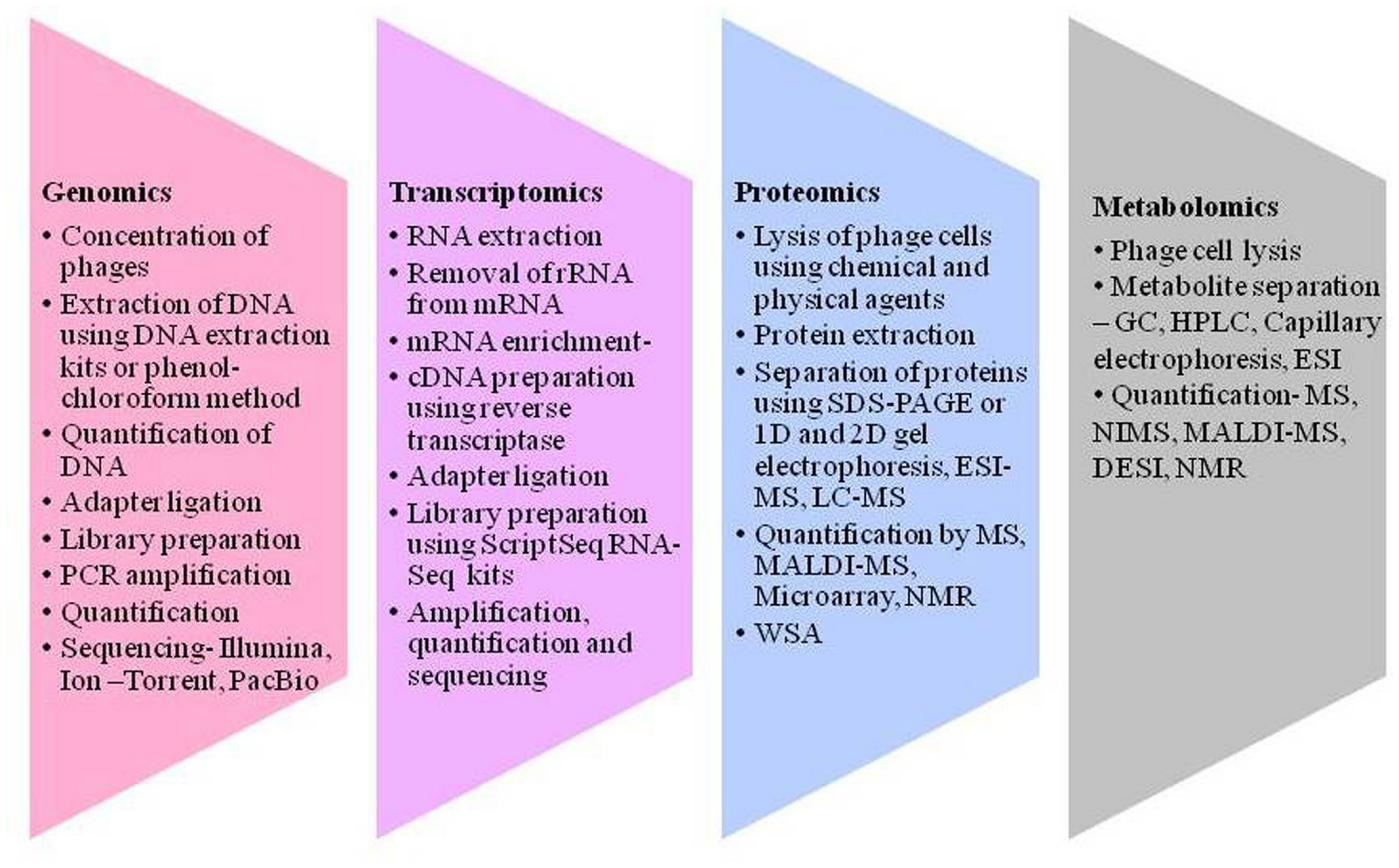
**Different techniques to gain an insight into virosphere**. Genomics includes concentration of phages, DNA isolation, quantification, and sequencing. Transcriptomics includes processing of RNA converting it to cDNA and sequencing. Proteomics encompasses protein extraction, separation and quantification using several tools like sodium dodecyl sulfate-polyacrylamide gel electrophoresis (SDS-PAGE), electron spray ionization—mass spectroscopy (ESI-MS), liquid chromatography–mass spectrometry (LC-MS), matrix-assisted laser ionization and deionization (MALDI)-MS and nuclear magnetic resonance (NMR), and Whole phage shotgun analysis (WSA). Metabolomics refers to metabolite extraction separation and quantification in a given time and different metabolites can be analysis using different tools like nanostructure initiator MS (NIMS) and desorption electron spray ionization (DESI) for the understanding of bacteriophage and its interactions.

The interplay between host and phage particle initiates as soon as the phage recognizes specific receptors on the bacterial cell wall. The tail proteins of phage particle recognize the receptor protein(s) of bacteria and inject own DNA into host cytoplasm choosing either lytic or lysogenic lifecycle. Once the phage DNA is inserted into the bacterial cell, the cell is termed as a “virocell” carrying virus auxiliary metabolic genes (vAMGs), which are believed to augment the metabolic potential of the host during infection process as shown in Figure 1 (Rosenwasser et al., 2016). The phages acquire new genes into their genomes by interactions with the host genome in order to replicates in the host cells. The bacterial genes that attach near to the prophage attachment site, suggests the genes were acquired by inaccurate prophage excision. Some novel genes can similarly be transmitted into the interior part of the genome by some unexplained mechanism (Juhala et al., 2000). However, these genes may be autonomous transcripts or repressed prophages that provide benefit to hosts (Brüssow et al., 2004). Horizontal gene transfer (HGT) by these phage particles from one host to another host genomes, results in an increased microbial diversity (Dutta and Pan, 2002; Weinbauer and Rassoulzadegan, 2004). Thus, the interaction between phage and host chiefly emphasizes the structure of microbial communities (Rohwer and Thurber, 2009). Some genes derived by phage also aid in nutrient cycling and gear up the biogeochemical cycles on Earth. Furthermore, phages have a crucial aspect in host mortality, carbon cycling (Breitbart et al., 2004) and nutrient cycling (Suttle, 2007). Also, microbial lysis by phage infection has significance in bacterial population control and the debris of these dead microbes act as a food source in the food web of the environment (Sime-Ngando and Colombet, 2009), thus involved the cycling nutrients (Figure 1). Phages are thus accounted as an application to limit bacterial pathogens and multi-drug resistant organisms in the environment by the mechanism of specifically lysing the bacterial hosts (Parmar et al., 2017). Despite an immense abundance and diversity of phages and their reimbursement in the global webs, molecular knowledge of phage-host interactions is missing. In the era of NGS, employing genomics, single cell genomics, transcriptomics, proteomics, and metabolomics can be a smart attempt to understand the interaction among the phages and their bacterial hosts (Figure 2). A review of the literature has been solicited to confer the claims of omics approach in phage research (Table 1).

**Table 1:** **Applications of omics approach for understanding bacteriophage–bacterial host interaction**.

**Sr. No**.	**Bacteriophage**	**Bacterial Host**	**Omics**	**NGS platform and tools**	**Applications**	**References**
1	*E. coli* bacteriophages lambda and T4	*E. coli*	Single virus genomics	FACS, MDA, and 454 GS FLX	Viral diversity, evolution, adaptation, and ecology	Allen et al., 2011
2	Rumen bacteriophages	Rumen bacteria	Metagenomics	Roche GS FLX	Phage-bacterial relationship	Berg Miller et al., 2012
3	Ø11b	*Flavobacterium*	Genomics and Proteomics	Shotgun sequencing, PAGE and MALDI-TOF-MS	Genome and proteome analysis	Borriss et al., 2007
4	Marine viruses	Marine bacteria	Metaproteomics	HiSeq-2000	Protein profile study	Brum et al., 2016
5	ØvB_CcoM-IBB_35	*Campylobacter coli*	Genomics and Proteomics	Pyrosequencing, SDS-PAGE and MS	Genomics and protein genes	Carvalho et al., 2012
6	Six different phages	*P. aeruginosa*	Metabolomics	TOF-MS	Alterations in host metabolism during infection	De Smet et al., 2016
7	Ø 812	*Staphylococcus*	Proteomics	1-DE, 2-DE, and MALDI-TOF-MS	Proteome analysis	Eyer et al., 2007
8	A4 Mycobacteriophage Kampy	*M. smegmatis*	Transcriptomics	RNA-Seq and MS	Profiling active genes	Halleran et al., 2015
9	Mycobacteriophage	*Mycobacteria*	Metaproteomics	ABI 3730 or ABI3100	Protein family studies	Hatfull et al., 2006
10	Skin virome	–	Metagenomics	Computational approaches	Exploring the viral dark matter	Hurwitz et al., 2016
11	MS2 bacteriophage	*E. coli*	Metabolomics	Flux balance analysis	Host-pathogen interaction	Jain and Srivastava, 2009
12	vB_EcoP_SU10	*E. coli*	Genomics and proteomics	Roche/454 and Nano LC-MS/MS analysis	Genomics and protein cataloging	Khan Mirzaei et al., 2014
13	RNA Bacteriophages		Metagenomics and, transcriptomics	Various based on subjects studied	Study novel RNA phages and their transcripts	Krishnamurthy et al., 2016
14	ØrV5	*E. coli* O157:H7	Genomics and proteomics	Clone library and primer walking and MS/MS	Using phages as analytical tools	Kropinski et al., 2013
15	Marine surface viruses	Marine surface bacterioplankton	Single cell genomics	Various	Host-virus interaction	Labonté et al., 2015
16	ØKMV	*P. aeruginosa*	Proteomics	SDS-PAGE, WSA and LC-MS	Structural proteins	Lavigne et al., 2006
17	Mycoviruses	Plant pathogenic fungi	Metatranscriptomics	RNAseq- Illumina	Diversity of mycoviruses	Marzano et al., 2016
18	ØvB_YecM_ΦR1-37 (ΦR1-37)	*Yersinia enterocolitica*	Transcriptomics	Whole genome transcription	Host-virus interaction	Leskinen et al., 2016
19	Cyanomyo-virus P-SSM2	marine *Cyanobacterium*	Transcriptomics	whole genome transcription	Host transcriptional response on infection	Lin et al., 2016
20	ØDp-1 and ØCp-1	*S. pneumoniae*	Proteomics	AP/MS	Interactions between bacteria and phage	Mariano et al., 2016
21	T5-like Bacteriophage	*E. coli* O157:H7	Genomics, Proteomics	454 Technology by GS FLX, SDS-PAGE, and MALDI TOF MS	Check for potential as biocontrol agent	Niu et al., 2012
22	ΦMSP	*S. aureus*	Proteomics	SDS-PAGE and 2DE	Proteomic characterization	Sangha et al., 2014
23	Halophilic viral communities	Halophiles	Metatranscriptomics	Microarray, DGGE, and ABI PRISM 310 DNA Sequencer	Metatranscriptome analysis of halophilic viruses	Santos et al., 2011
24	ΦCP39O and ΦCP26F	*C. perfringens*	Genomics and proteomics	Pyrosequencing, 2DE, and MALDI-TOF-MS	Prediction of genome and proteome	Seal et al., 2011
25	ΦTMA	*Thermusthermophilus*	Genomics and proteomics	ABI 3700 Sequencer, PFGE, and SDS-PAGE	Genomic and proteomic characterization	Tamakoshi et al., 2011
26	Different phages against *P. aeruginosa*	*P. aeruginosa*	Proteomics	Affinity purification with MS	Identification of hypothetical proteins	Van den Bossche et al., 2014
27	MC1061 (Φ24B)	*E. coli*	Transcriptomics	Whole-genome shotgun pyrosequencing	Altered functions of host after phage infection	Veses-Garcia et al., 2015
28	ØwV8	*E. coli* O157:H7	Genomics and proteomics	Pyrosequencing, MALDI-MS, and QqTOF	Genomic and proteomic analysis	Villegas et al., 2009
29	ØPhV1 type A and ØPhV1 type B	*Cyanobacteria*	Metagenomics and metatranscriptomics	HiSeq-2000	Interactions between bacteria and phage	Voorhies et al., 2016
30	ØvB_BpuM_BpSp	*Bacillus*	Proteomic analysis	HPLC-ESI-MS/MS	Check a particular activity of protein	Yuan and Gao, 2016
31	ØPaP3	*P. aeruginosa*	Transcriptomics	Microarray, RT-qPCR	Interactions among bacteria and phage	Zhao et al., 2016

*1-DE, 1 dimensional electrophoresis; DGGE, denaturing gradient gel electrophoresis; FACS, fluorescence-activated cell sorting; LC-MS, Liquid chromatography–mass spectrometry; MDA, multiple displacement amplification; MALDI-TOF-MS, matrix-assisted laser desorption/ionization time-of-flight mass spectroscopy; PAGE, polyacrylamide gel electrophoresis; PFGE, pulsed field gel electrophoresis; RT-qPCR, Real-time quantitative polymerase chain reaction; RNA-Seq, RNA-Sequencing*.

## Application of genomics to reveal phage diversity

Owing to the insufficiency of viral database, there is more than 90% viral dark matter (Hurwitz et al., 2013, 2015). Additionally, the absence of a biomarker gene among phages leads to sequence the whole phage genome for its understanding (Thurber et al., 2009). The genomics of phages would elucidate the genetic composition and putative functional role in the environment. Phage metagenomics would furthermore assist in determining the diversity of phages in a community and reveal novel genes demonstrating phages to be the most diverse beings on the globe (Edwards and Rohwer, 2005). Subsequently, interpretation of functional channels of bacterial viruses would illuminate host-phage interactions (Brum et al., 2015).

**Figure 3:**
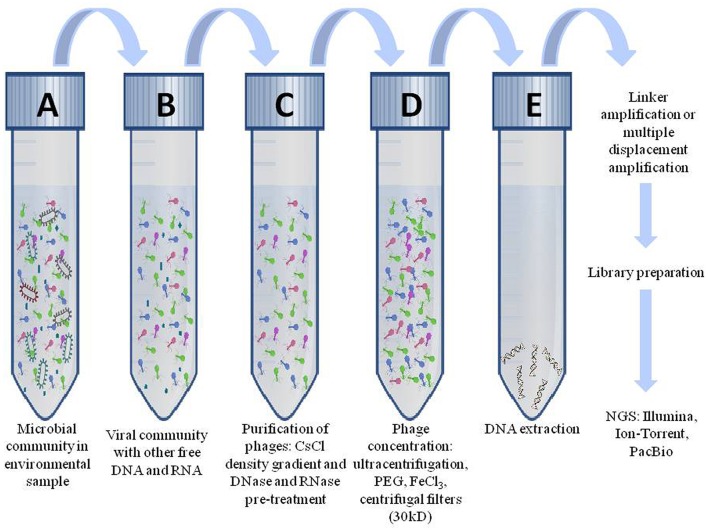
**Workflow to study the genomic content of bacteriophages**. Genomics of phages initiates by filtering phage particles from microbial community **(A)** through 0.22 μ filter which results in **(B)** virus particles containing residual DNA and RNA of other microbial communities. **(C)** Purification of virus particles from residual DNA and RNA are removed by CsCl density gradient method. **(D)** Phage particles are concentrated using polyethylene glycol or ultra-centrifugation. **(E)** Extraction of viral DNA uses kits or conventional methods. The DNA is amplified and libraries are prepared and subsequently sequenced them.

A typical genomic experiment begins with isolation of genomic DNA of virus particles (Figure 3). The primary step is filtration of the sample through 0.22 μm filters for elimination of bacterial constituents and other contaminations. Samples are then concentrated by ultracentrifugation or polyethylene glycol precipitation (Helms et al., 1985) and subjected to DNase and RNase treatments to exclude residual genomic material from any contaminant bacteria that may pass through 0.22 μm filter. This treated sample would include only virus particles which can be cleaved and their genome can be extracted using kits or standard methods (Adhikary et al., 2014). In order to examine with NGS platforms, DNA is fragmented, ends are repaired and are ligated with adaptors (Holmfeldt et al., 2013). Finally, fragmented DNA library is cleaned and amplified through PCR as well as is quantified and sequenced. Several sequencing platforms are available such as Ion Torrent, Illumina, PacBio which are preferred as per the requirement of their read length, coverage, paired reads, insert size, accuracy, error rates, sequencing yield, run time and sequencing cost (Quail et al., 2012). To reduce chances of bacterial contamination in the library, a section of DNA is PCR amplified for 16S rRNA genes, and if bands are detected, it conveys the presence of host contamination. In the instance of lesser viral DNA yields, amplification using multiple displacement amplification (MDA) can be performed, but it may generate chimeras (Yilmaz et al., 2010). Apparently, amplifications using linkers may depict impartial viromes (Duhaime et al., 2012; Hurwitz et al., 2015). The sequences acquired by sequencing are developed for data filtering and the sequence reads that passes quality check, is mapped to reference genomes or assembled *de novo*. If the sequence of contaminating host is furthermore present in reads even after purifying the sample, it can be distinguished by comparing reads to reference bacterial genome or 16S rRNA database (Hurwitz et al., 2013). For annotation of viral genomes, a database such as NCBI non-redundant nucleotides can be used. ORFs can be determined and annotated using CyVerse (Goff et al., 2011) in the PCPipe application through the iVirus project (Hurwitz et al., 2014).

Bioinformatic tools mine enormous volume of sequence data to determine common patterns that govern microbes in an ecosystem. Viral diversity can be estimated using PHACCS toolkit (Angly et al., 2005). To decode the correlation between virus community and environmental factors, an application called Fizkin by CyVerse cyber infrastructure iVirus project selects 300K reads arbitrarily from viromes and examines it using Jellyfish that generates a matrix of shared sequence counts between each virome pair. This matrix uses an input file for Bayesian network analysis resulting in a table of the relevance of environmental factor that determines the diversity of virus and a social network graph (Hurwitz et al., 2014, 2016). This will assist in ecological profiling of viral communities without requiring assembly and annotation. To elucidate sequence matches with a reference database, BLAST is regularly employed along with MG-RAST (Glass et al., 2010), MetaPhyler (Liu et al., 2010) or CARMA (Gerlach et al., 2009). For a taxonomy of viruses, MEGAN (Huson et al., 2007) software can be used whereas Hidden Markov Models, e.g., HMMER (Finn et al., 2011) are applied to match Pfam or KEGG domains. To find specific viral species present in metagenome, k-mer based algorithms such as CLARK (CLAssifier based on Reduced K-mers) (Ounit et al., 2015), USEARCH (Edgar, 2010), KRAKEN (Wood and Salzberg, 2014), and NBC (Naïve Bayes Classifier) (Rosen et al., 2011) have been applied. Sometimes, whole host genome can be observed in viromes when gene transfer agents (GTAs cluster) have filtered along with virus-like particles (Roux et al., 2013b). GTAs and sporadic contaminations can likewise be recognized using software CLARK (Ounit et al., 2015). Alignment of sequences with reference bacteria genome may reveal a prophage viral element using “recruitment plot” in the bacterial genome. Some of the bioinformatics tools adapted for prophage detection include ACLAME, Prophinder, PHAST and PhiSpy which can serve in confirming phage annotation (Akhter et al., 2012).

Along with elucidating diversity and taxonomy of phages, establishing the origin of genes (bacterial or viral) is vague. This ambiguity occur because of vAMGs which incorporates (enhancing cell metabolism in the host) into host cells or some viruses may also uptake some bacterial genes near the prophage excision site. Nevertheless, during integrating into host tRNAs, phages carry an attachment site (attP) which denote a definite match of a host tRNA gene. Example, integrase gene and an attP site (53 bp) of the *Prochlorococcus* phage P-SS2 is a precise analogue of the host tRNA (attB, 36 bp) of *Prochlorococcus* MIT9313 (Sullivan et al., 2009). Such phages that display a putative attP site and an integrase identical to a host tRNA gene fragment are suggestive of a host-phage association (Mizuno et al., 2013). Metagenomics serves to find diversity among phages but knowledge about interaction among phage and host is relatively scanty. By analyzing the spacers in CRISPR to phage metagenomes, the bacterial host of phage (Dutilh et al., 2014) and phage-host interactions can be deduced (Anderson et al., 2011; Berg Miller et al., 2012; Edwards et al., 2016). Characterizing these constraints is requisite to develop our insight about bacterial-phage co-evolution.

### Single cell genomics

Apart from metagenomics, attempts have been instigated to investigate only an individual isolate in detail reinforcing our perception of the mechanisms of a specific cell rather than the influences of the entire population. Recently, single cell genomics (SCG) has been promoted to infer the phage genome which is present in or on the surface of host cells in a particular niche without culturing (Lasken and McLean, 2014) and facilitates in assuming sole genetic and metabolic profiles of uncultivable microbes. SCG helps in understanding the interplay between the phage and their host and can ascertain phage genome in a bacterial host cell. To isolate a single cell from an environment, techniques such as flow cytometry (Podar et al., 2007) and micromanipulation (Ishøy et al., 2006) have demonstrated to be advantageous. To sort a single cell precisely, a fluorescence-activated cell sorter (FACSAria™) with a forward scatter photomultiplier tube (PMT) has been adopted to simplify accurate detection and high-resolution entry of single cell (Picot et al., 2012). Confocal laser scanning microscopy has been applied to support a single phage separation stained by fluorescent dyes lodged into agarose (Luef et al., 2009). Multiple displacement amplification (MDA) (Hosono et al., 2003) utilizes an advanced properties of phi29 DNA polymerase which intensifies a microbial genome at million-fold, sufficient for sequencing using any of the available sequencing platforms.

Interpretation of viral diversity has become easier after the expansion of single virus genomics (Allen et al., 2011) while attending one virus at a time. New computational challenges to analyze the outcomes of SCG using bioinformatics tools have emerged, reflecting the vast opportunity to figure out the *in-situ* phage-host communications. Several bioinformatics tools have been in practice for the classification of prophages-pathogenicity islands such as PIPS (Soares et al., 2012) and HGT- using Alien Hunter (Vernikos and Parkhill, 2006), but these tools seem weak when studying novel phages because of a deficiency of genomic sequences in the viral public database. Because of this constraint, semi-continuous and partial SCG sequences in the database do not allow the accurate identity of isolates (Kalisky and Quake, 2011). However, SCG provides cytoplasmic insights during various interactions with phages like lysogeny, lytic infections, chronic infections and unspecific attachments (Allen et al., 2011). For distinguishing between these synergies, sequences have been examined for integration of phage into the host DNA, portion of phage and host DNA was measured for the speed of single cell MDA reactions and comparisons have been made between the coverage depth of phage and host contigs (Labonté et al., 2015). Phages infecting previously unknown hosts have been discovered in the marine environment using this technique (Roux et al., 2013a, 2014; Labonté et al., 2015).

Non-specific amplification or distortions in the single genome may be a reason for the loss of data, but approximately 90% of DNA can be retrieved using SCG (Rodrigue et al., 2009). A newly developed technology called Hi-C sequencing determines closely arranged genome sequences, like virus-bacterial host genomes within an individual cell (Beitel et al., 2014). The concept of this facility includes genome cross-linking using formaldehyde and a restriction excision, followed by re-ligation of sequences using ligases in a dilute condition that support ligation events between cross-linked DNA fragments, conforming the pairs to each other that were originally in close contiguity (van Berkum et al., 2010). This technique can be adapted to phage-bacterial host communities to figure out close entity while they have been successfully operated for various microbial studies (Beitel et al., 2014; Burton et al., 2014).

Oxford Nanopore sequencing has been utilized where individual DNA molecule is directly sequenced without amplifying or labeling genome with chemical or using visualization tool to recognize the chemical label (Branton et al., 2008). Nanopore sequencing works on the principle that when a voltage is applied to a nanopore imbibed in a conducting liquid, electric current can pass through the nanopore. This electric current is highly responsive to nanopore size and shape such that indeed a single passage of DNA nucleotide pass through nanopore could affect an alteration in the current. The magnitude of current differs based on the type of nucleotides (A, T, G, or C) passing through the nanopore. Thus changing in current corresponds to the precise sequence of a DNA stretch. Viral pathogens have been examined using Nanopore technology (Greninger et al., 2015). Concurrently, MinION sequencer has similarly been recommended which is incredibly rapid, smaller in size, produce 200 kb long reads with high accuracy as well as it has been used to study lambda phage DNA (Mikheyev and Tin, 2014).

**Figure 4:**
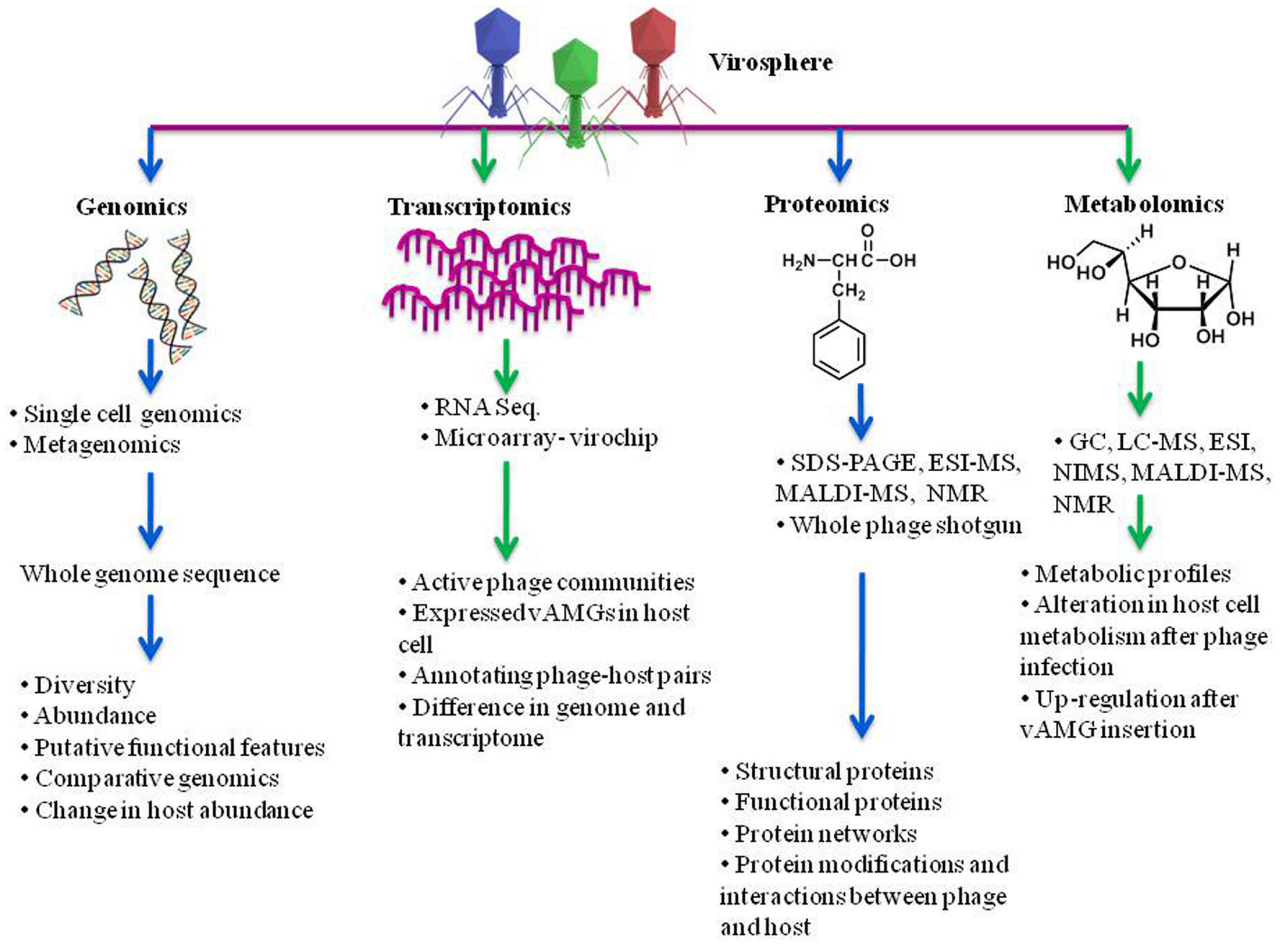
**Understanding phage diversity, community interactions and chemical profiles using meta-omics approach**. Genomics elucidates the phage diversity, abundance, probable functional features while transcriptomics gives an insight about the actively expressed genes in a community. Proteomics suggests the phage structural proteins, its functions and the proteins responsible for interaction between phage and host. Metabolomics advocates the metabolites produced by hosts in presence and absence of phage infection, alterations in regulation and metabolic profiles after infection. SDS-PAGE, sodium dodecyl sulfate-polyacrylamide gel electrophoresis; ESI-MS, electron spray ionization–mass spectroscopy; LC-MS, liquid chromatography–mass spectrometry; MALDI-MS, matrix-assisted laser ionization and deionization; NMR, nuclear magnetic resonance; NIMS, nanostructure initiator MS.

Metagenomics and SCG technologies can be strongly adapted to illustrate the exact identity and diversity of phages in an environment which can guide across the dark matter of viral ecosystem (Figure 4). Along with investigating the diversity of phages in an environment, to succumb with a coherent outline of their functional aspect in an ecosystem, it becomes imperative to deduce mechanisms underlying transcriptions of phages. Hence, transcriptomic studies provide knowledge about functions of active genes in given condition.

## Employing transcriptomics to study active phage function

Transcriptomics provides a measure to investigate the active microorganisms within a community at a specific time and under a definite array of conditions. Study of the transcriptome is critical to analyze molecular constituent of phages and to understand genome function during a distinct period or situation such as development or infection state. The principle objectives of transcriptomics include recording transcripts of all species including mRNAs, non-coding RNAs and small RNAs, to estimate transcriptional organization of genes in terms of 5′ end and 3′ end, gene splicing and post-transcriptional modification as well as to demonstrate varying activity of each gene under different conditions (Bikel et al., 2015). Several technologies have been developed to inspect and determine transcriptomes, such as DNA hybridization technique, DNA microarray, cDNA-amplified fragment length polymorphism (cDNA-AFLP), expressed sequence tag (EST) sequencing, serial analysis of gene expression (SAGE), massive parallel signature sequencing (MPSS), and RNA-seq (Mutz et al., 2013). DNA hybridization employs fluorescently labeled cDNA to hybridize with DNA templates on microarray chips. However, this tool possesses some reserves as it relies on the hitherto studied genome sequences, high background levels for cross-hybridization and a smaller detection range. Phage meta-transcriptomics may present few difficulties as phages are incredibly diverse and their database is considerably less. Also, the availability of RNA, especially mRNA may be in a rather less volume because of inactive phase when phage is not in association with bacterial hosts. Thus, this may lead to challenges in isolating and enriching mRNA for sequencing.

A typical transcriptomics analysis using NGS includes isolation of the total RNA from the virus particles, depending on the RNA to be sequenced (mRNA, lincRNA or microRNA). Initially, the bacterial fractions are separated and purified from the phage particles. DNase and RNase treatments are administered to filter phage particles from any free DNA or RNA of bacteria. The RNA is thus extracted from virus particle using RNA extraction kits such as RNeasy mini kit. Selective elimination of rRNAs can be achieved using rRNA removing kits or using probes complementary to the rRNA region that is attached to magnetic beads. The mRNA can be enriched by magnetic bead capture method of rRNA, preferential polyadenylation of mRNA or preferential digestion of rRNA through enzymes. The cDNA are synthesized using random hexamers or oligo (dT) primers or priming with poly dT primers after polyadenylation. For amplification, RNA polymerase (Ozsolak and Milos, 2011) or MDA (Gonzalez et al., 2005) or emulsion PCR is/are performed (Tang et al., 2009). The 5′ and/or 3′ ends of the cDNA are then repaired along with adapter ligation, following library cleanup, amplification, quantification and sequencing of the library. Single-end or paired-end libraries can be prepared using kits like ScriptSeq RNA-Seq library preparation kit (Illumina, San Diego, CA) and can be sequenced on platforms such as Illumina HiSeq2500. Sometimes, conversion of RNA into cDNA introduces bias into the quantification of transcripts, thus a semi-direct sequencing of RNA by-passing the synthesis of cDNA has been established (Hickman et al., 2013).

The bioinformatics analysis of raw data retrieved by transcriptome sequencing uses reference genes and genomes to map against the raw reads or performing a *de novo* assembly for unreported transcriptomes. Mapping of transcripts against reference genome would confer taxonomy and function of active phages. Mapping the active functional pathways would expound the up-regulated, down-regulated or unaffected genes of phage during development or infection cycle. The same can be advised for the bacterial host during infection by a phage. The transcriptome reads which are short can be assembled *de novo* using several softwares such as Trinity. Efficiency and sensitivity of the software are exceptionally promising in procuring full-length transcripts (Ghaffari et al., 2014). The assembled contigs that are obtained by *de novo* or reference-based assembly can be equated with the NCBI viral reference amino acid sequence database using USEARCH (Edgar, 2010). Moreover, the virus annotated hits can be compared with NCBI non-redundant database using BLASTX. Bowtei software can be used to calculate sequence read number and coverage depth (Langmead et al., 2009). Alignment of reads can be prepared using software called MUSCLE (Edgar, 2004) and for constructing neighbor-joining tree, MEGA software can be employed as uses for the bacterial analyses (Tamura et al., 2013).

Several phage meta-transcriptomics studies have been conducted in an attempt to analyze active phage communities. Studies have revealed the effectiveness of phage metagenome for constructing templates in the microarray (Virochip) to annotate and identify the sample (Santos et al., 2011). The total RNA extracted from the sample can be converted to cDNA, labeled and allowed to hybridize with the virochip (Santos et al., 2012). A combination of metagenomics and meta-transcriptomics study would specifically determine active phages in an environment in which phage transcripts may vary as compared to their genomic abundance (Lim et al., 2013). There can be a case when a particular set of family of genes remain less abundant in metagenomic analyses whilst those genes may be remarkably active in meta-transcriptomics dataset and/or vice versa (Franzosa et al., 2014). This insinuates that performing only a metagenomic study may not be a perfect snapshot of functional active genes in a metagenome. To overcome the tedious isolation of viral mRNA from total mRNA, SCG can be conducted along with microarrays to designate the phage-host systems without cultivating them (Santos I. M. et al., 2015). In another study, phage-host pairs have been investigated in which a fosmid viral metagenomic library was constructed and immobilized on microarray “virochip,” along with them. The genomes of uncultured bacterial host cells can be sorted by fluorescence-activated cell sorting (FACS) followed by amplification via MDA. Single host cells were hybridized on virochip, and the host cells and immobilized phages with positive results were sequenced (Martínez-Garcia et al., 2014). With this new technique, advancements toward the discovery of phage-host interactions arise in current decade (Santos F. et al., 2015). Moreover, meta-transcriptomics based enzyme discovery from phages can assist in utilizing novel enzymes with specific enzymatic characteristics for the industries and scientific communities (Schoenfeld et al., 2010).

Transcriptomics can be employed to analyze the influencing of a phage on the bacterial host after the phage infection. One such response includes induction of Shiga toxin production and acid resistance in *E. coli* by Shiga toxigenic phages (Veses-Garcia et al., 2015). Studies confirm the fact that host genes get differentially expressed after the phage infection such as a phage “PaP3” had a down regulatory impact on host transcriptional regulators and it proved early genes of phage affected strongly by regulation of hosts (Zhao et al., 2016). This feature of phages can be promoted for formulating a phage therapy. Transcriptomics studies of phage during infection of the host can serve an insight of sequence of transcriptional events, such as initial phase consisting of gene metabolism, DNA synthesis, and regulation genes, is accompanied by a prolonged phase of structural and lysis genes (Halleran et al., 2015). These views can yield information about vAMGs which alters metabolic functions of bacterial host after phage infection. During late phase of phage infection, several up-regulating mechanisms have been observed in bacterial gene expression including stress response and stability of membranes (Leskinen et al., 2016). Additionally, enrichment of ATP synthase and ribosomal protein genes have been revealed during phage infection of phosphorous starved *Cyanobacterium* host (Lin et al., 2016). With further advancement and a few drawbacks, transcriptomics gives an insight of the phage-host interactions and evaluates the regulatory mechanism in bacterial hosts by phage and/or vice versa (effect of host interactions on phage regulation) which are noteworthy for developing phage therapy and comprehend novel phage antimicrobial compounds.

## Understanding the proteomic profile of phages

A proteome can be represented as a set of all expressed proteins in a cell, tissue or an organism (Theodorescu and Mischak, 2007). Proteomics is a methodology for the characterization of genetic data in a cell or an organism via protein pathways and networks (Petricoin et al., 2002) and for distinguishing the functional implication of proteins (Vlahou and Fountoulakis, 2005). It focuses at cataloging protein expression profiles at a specific period, in a definite location of the cell and as a response to foreign stimulations. It is applied to design a plot of protein networks which can be used to demonstrate interaction among protein in an organism (Corpillo et al., 2004). It provides an estimate of occurrence, quantity and modified state of proteins in an environment in a significant-throughput method.

Genome and transcriptome analysis evaluate the indirect functional profile of a cell or a community whereas proteomic reveal a direct estimate of functional activity of a cell (Schwanhäusser et al., 2011). Abundance profiles of proteins can be plotted using comparative metaproteomics, while the reduction or increase in the quantity of some proteins may signify a distinct purpose in an organism or during particular situations of phage infection on the bacterial host (Sangha et al., 2014). The post-infection protein expression changes can be classified as (1) function which alters rapidly on phage-infection, but can get reverted back (2) variations that develop gradually and persist consistent or cannot revert back, and (3) alterations that appear abruptly and are maintained for a longer term.

Developments in next generation tools have drastically enhanced quantification and identification of proteins (Schleicher and Wieland, 1978). The proteomic analysis commences with phage concentration accompanied by lysing phage using physical and chemical agents, consequently releasing phage proteins (Figure 2). The concentration of proteins can be measured using Bradford's method (Bradford, 1976) or can be denatured using urea (Lavigne et al., 2006) or can be digested by trypsin (Borriss et al., 2007). Several approaches and facilities have been in practice for proteomic studies (Chandramouli and Qian, 2009), however, employing some tools such as a mass spectrometer (MS) and protein-chips (microarray) have significantly contributed in the field (Horgan and Kenny, 2011). Proteins have earlier been detected and quantified using enzyme-linked immunosorbent assay (ELISA) and Western blot where proteins were initially separated by sodium dodecyl sulfate-polyacrylamide gel electrophoresis (SDS-PAGE) (Lavigne et al., 2006). Studies have been performed to understand phage proteins using MS after separating by 1D and 2D PAGE (Clement et al., 2013). Additionally, mass-spectrometry-based techniques such as matrix-assisted laser desorption/ionization (MALDI-MS) (Borriss et al., 2007) and electron spray ionization (ESI) (Carvalho et al., 2012) have been established for analyzing various proteins of phages. Recently, fluorescence 2D differential gel electrophoresis has been employed to distinguish between amounts of human lymph and plasma proteins (Clement et al., 2013). Structural proteomics can interpret the structure of proteins thereby determining the functions of novel genes. Lavigne et al. (2006) described structural proteome of phiKMV, a lytic bacteriophage of *Pseudomonas aeruginosa* using SDS-PAGE, LC-ESI-MS/MS, and GC-MS. Nuclear magnetic resonance (NMR) (Horgan and Kenny, 2011) and X-ray crystallography (Drulis-Kawa et al., 2012) can be employed to investigate the interaction between phage-binding protein and receptor site on the bacterial host (Sundell and Ivarsson, 2014).

Protein analysis using MS requires a prior separation of the sample either by 2D-gel electrophoresis (Renesto et al., 2006) or isotope-coded affinity tag (ICAT) labeling (Weston and Hood, 2004), accompanied by digestion into peptides and separating peptides using LC. Microarrays can be applied for assorting protein interaction with DNA, protein or ligands. Protein microarray technique can be exploited in the analytical study to check for presence/absence of a distinct protein in a sample (biomarker detection during phage infections) or for defining function (Uzoma and Zhu, 2013). When phage proteins are immobilized on a microarray chip, it can be applied to probe for complementary bacterial host receptors that bind with phage recognition proteins (Santos F. et al., 2015). Reverse-phase protein microarray can serve as a comparative protein profile in case of phage-infected and uninfected bacterial host (Haider and Pal, 2013). Thus, correlative examination of proteome and genome provide an interpretation of the post-translational modifications.

Functional identification of hypothetical phage proteins is performed using MS analysis after affinity purification of host protein mixtures (Van den Bossche et al., 2014). MS/MS spectra can be interpreted using SEQUEST (http://fields.scripps.edu/sequest/) or Mascot (Matrix Sciences) and classifying using DTASelect and Contrast softwares (Tabb et al., 2002). Proteomic phage display techniques are similarly employed to identify target proteins and consensus motifs (Sundell and Ivarsson, 2014). Whole phage shotgun analysis (WSA) is a recently developed technique for protein analysis using NGS platform. It is a culture-independent technique which offers annotation of proteins associated with phages. WSA combines all structural proteins separated on the basis of mass and charge before identification (Lavigne et al., 2006). After separation, the data can be annotated to open reading frames (ORFs) by aligning with reference protein sequences using BLASTP. HHpred is another tool for assigning the protein structure (Hildebrand et al., 2009). The function and evolution of identified proteins can be determined by program COGnitor (www.ncbi.nlm.nih.gov/COG) and InterProScan to find conserved domains (Eyer et al., 2007). When a predicted protein does not match along known proteins from the database, protein clustering can be developed for the comparative analyses to assess the protein diversity (Hurwitz et al., 2013; Brum et al., 2016). Some software can extract the data from MS and microarray and decipher protein identification using databases such as UniProt (http://www.uniprot.org/), PROSITE (http://prosite.expasy.org/), Pfam, Conserved Domain and PDB databases. Thus, with an advent in high-throughput proteomic technology, analytical tools, bioinformatics software and database, research on proteins have emerged as an easy task to elucidate protein matter in an environment.

## Cataloging the metabolome of virosphere

The breakdown products of metabolism or intermediates involved in the process of metabolism are termed as metabolites. Metabolites can be (1) primary- which are precisely involved in process of metabolism or (2) secondary- which may not directly take part in the growth of an organism. The metabolome of an organism corresponds to a set of metabolites including hormones, intermediates, signaling and secondary molecules in a particular cell, tissue, organ or an organism (Griffin and Vidal-Puig, 2008; Jordan et al., 2009). To explain the physiology of a particular cell, the study of metabolites is very substantial as every cell possesses a specific metabolic catalog which can influence the accurate implication of function of a cell or an organism (Nicholson and Wilson, 2003; Zhang et al., 2016). These are results of gene transcriptional and translational mechanisms which remain exceptionally complex, hence variations in metabolites intensify as compared to variations among transcriptome and proteome.

Various approaches have been established for separation and detection of metabolites, chiefly when metabolites are of higher molecular mass. The segregations of metabolites can be carried out using gas chromatography (GC) and high performance liquid chromatography (HPLC), capillary electrophoresis, electron spray ionization (ESI) accompanied by GC, atmospheric-pressure chemical ionization (APCI) on the ground of characteristics of metabolite to be processed (Alonso et al., 2015). Detection of separated metabolites have furthermore been attainable by using nanostructure-initiator MS (NIMS), MALDI-MS, secondary ion mass spectrometry (SIMS), desorption electron spray ionization (DESI), and NMR (Drexler et al., 2007; Cornett et al., 2008; Wiseman et al., 2008; Greer et al., 2011). Statistical tools are additionally applicable for the evaluation of elicited data such as XCMS (Patti et al., 2013), MZmine (Katajamaa et al., 2006), MetAlign (Lommen, 2009), MathDAMP (Baran et al., 2006), and LCMStats^1^ (Gahlaut et al., 2013). The metabolic database is available in form of METLIN (Smith et al., 2005).

Metabolomics would serve in interpreting the significance of active phage community on the environment in real time. Based on distinct phases of the phage infection or metabolic profile of the host infected with a phage, the gene markers can be inferred. The modification in host-cell metabolism by phage-encoded genes (vAMGs) into the host genome, is described as a virocell amendment (Rosenwasser et al., 2016). Studying highly specific metabolic profiles of a virocell can improve in interpreting metabolic profile of vAMGs. Such comparisons were conducted to recognize host-viral interactions (Vardi et al., 2009, 2012; Fulton et al., 2014). Metabolomic analysis of phage interprets the influence of the vAMGs which is responsible for enhancing nucleotide biosynthesis (De Smet et al., 2016) via degrading host macromolecules such as DNA through catabolic pathways. The vAMGs encoded nucleases can generally degrade host DNA and encoded triglyceride lipase can degrade host triacylglycerols which yield energy and ultimately engages in the the formation of virus membrane (Malitsky et al., 2016). Example, ceramidase in *Mimivirus* helps in the catabolism of sphingolipids (Arslan et al., 2011). Thus, the vAMGs develop the metabolic potential of virocell through triggering novel enzymes which were not present in host machinery prior to phage infection (DeAngelis et al., 1997; Graves et al., 1999). The vAMGs can serve as a shunt between phage and their host by imparting several functional genes from one another especially assisting during stress conditions (Rosenwasser et al., 2016). These mechanisms illustrate unique attributes of gene products of phage that can mediate dynamics phage-host interaction, as an effect, shaping the microbial communities in an environment. Thus, biochemical composition and metabolic profile of bacterial hosts are greatly governed by phages and released metabolites in the environment influence the microbial food web (Miki et al., 2008). Cataloging the metabolome of phages can elucidate special phage-derived metabolites which usually act as decision making between lytic or lysogenic lifecycle in virocell. Study of metagenomic and metabolomic profiles can simultaneously determine whether the metabolites are encoded by the phage or the host. Furthermore, metabolic profiles of phage can aid in tracing a novel biomarker to recognize the nutrient source in biogeochemical cycles. Thus, the advents in the omics approaches utilizing NGS techniques bear a tremendous potential in exploring virosphere and thus the microbial world.

## Conclusions

Advancements in the field of NGS have facilitated the discoveries on the verge of a revolution in the course of microbial research. There has been a tremendous microbial data generated about the microbes present on Earth and their diversity and functional roles in regulating the ecosystem. Progress in interpreting the phage diversity and functions as well as the interactions among phages and their hosts are promising using the “omics” concepts. This would illuminate the function of phages in regulating microbial diversity by HGTs, governing the biogeochemical cycles, host population controls and determining the novel biomarkers. NGS will also nurture the upcoming phage therapy research for limiting MDR pathogens. With strong prospects in the field and developments in phage database, “omics” approach is witnessing a remarkable motive for a transformation in the yet uncultivable microbial research.

## Author contributions

KP, PD, and RS conceived and designed the work. KP, SG, and RS wrote the manuscript. PD, RK, and RS carefully checked the manuscript and corrected. All of the authors contributed to the discussion and approved the final manuscript.

### Conflict of interest statement

The authors declare that the research was conducted in the absence of any commercial or financial relationships that could be construed as a potential conflict of interest.

## Footnotes

^1^Lcmstats: LCMStats: an [R (programming language)] package for detailed analysis of LCMS data. http://sourceforge.net/projects/lcmstats.html.
